# Dynamic, anthracycline-induced chromatin alterations enable ALDH1 expression as a mechanism of inducible leukemia resistance development

**DOI:** 10.1007/s44313-026-00140-7

**Published:** 2026-05-13

**Authors:** Spiros A. Vlahopoulos

**Affiliations:** A’ Department of Pediatrics, University Research Institute of Maternal and Child Health and Precision Medicine, National and Kapodistrian University of Athens, Thivon & Livadias 8 Str., Athens, 11527 Greece

## Abstract

The evolution of acute myeloid leukemia (AML) cells toward developing drug resistance, which eventually leads to relapse, involves the convergence of their chromatin compaction status toward a pattern that is distinctive of “stem-like” cells and is conducive to increased expression of nuclear factor kappa B (NFκB). This phenomenon develops irrespective of the genetic mutations carried by the AML clones. Multiple lines of evidence therefore suggest that relapsed and refractory leukemia develops through alterations in chromatin, which perturb the normal cellular regulation of inflammatory and stress responses. In particular, during the development of acute leukemia phenotypes that resist drug treatment and give rise to relapse, malignant clones carrying different genetic alterations often converge into specific phenotypes with the apparent criterion being shared patterns of chromatin exposure. These shared patterns include genes involved in innate immune pathways that provide malignant cells with a selective advantage. Such a selective advantage is provided also by expression of cytosolic retinaldehyde dehydrogenases such as ALDH1A1, which was shown to characterize AML cells driving relapse, and which are resistant to chemotherapy.

In their recent paper in Hemasphere, Leonetti et al. showed that alterations in the chromatin-binding sites of transcription factors that regulate gene enhancers of cytosolic retinaldehyde dehydrogenases, aldehyde dehydrogenase 1 family member A1 (ALDH1A1) and 2, drive the expression of cytosolic aldehyde detoxification enzymes in response to anthracycline treatment [1].

They showed that high expression of ALDH1A1 correlates with prior chemotherapy exposure, intrinsic chemotherapy resistance, and poor survival outcomes. Anthracyclines induce ALDH1 transcriptional activation in AML cells, leading to enhanced enzymatic activity in drug-resistant models.

This transcriptional activation can be ascribed to anthracycline-induced histone acetylation changes at enhancers that activate the ALDH1A1 and ALDH1A2 loci. In particular, Jun proto-oncogene (c-JUN) and signal transducer and activator of transcription 3 (STAT3) bind to enhancers that mediate anthracycline-induced activation of ALDH1A1 and ALDH1A2. The significance of this phenomenon was shown via pharmacological inhibition of ALDH1 activity, which was synergistic with anthracycline (measured using the Bliss independence scores for cytotoxicity), and reduced the clonogenic potential of AML cells.

Confirming the above, a sequential administration of ALDH1 inhibitor dimethyl ampal thiolester (DIMATE) and daunorubicin resensitized anthracycline-refractory human AML cells KG-1a, xenografted in mice, improving in vivo efficacy.

ALDH1A1/1A2 inhibition disrupted cellular adaptive stress responses and synergistically enhanced the antileukemic efficacy of daunorubicin in resistant AML models, both in vitro and in vivo [1].

Recent longitudinal single-cell RNA sequencing analyses in refractory or early relapsed AML patients indicate the involvement of inducible STAT3 activity in ALDH1A1-regulating chromatin sequences in adaptive resistance; STAT3 was prominently upregulated within a subpopulation of quiescent stem-like cells exhibiting high chemotherapy-induced plasticity and a strong association with poor patient outcomes [2].

In solid tumors, specifically in non-small cell lung cancer, alterations in chromatin exposure driving the induction of dormancy and reactivation of lung cancer cells following cisplatin chemotherapy were investigated using transposase-accessible chromatin sequencing; STAT3 and JUN were key transcription factors showing enriched motifs during dormancy and reactivation [3].

STAT3 and JUN belong to the broader network of transcription factors that regulate cellular transitions from stress to inflammation and the subsequent return to homeostasis, to a certain extent also through interactions with the transactivator NFκB, and thereby enable pivotal chromatin alterations such that the cellular phenotype is affected; this radical event is referred to as a change of “cellular identity” [4]. The regulatory transitions that pivot this process also include the activation of STAT3 and JUN [5].

Previously, single-cell RNA sequencing of paired drug naïve and resistant AML patient samples showed that transcriptional plasticity drives stable epigenetic resistance. Lysine-specific demethylase 1 (Lsd1) inhibition could overcome stable epigenetic resistance by facilitating the binding of the pioneer factor, Pu.1, and the cofactor, interferon regulatory factor (Irf8), to nucleate new enhancers that regulate the expression of key survival genes. This enhancer switching redistributes transcriptional coactivators, including bromodomain-containing protein 4 (Brd4) [6]. Cytarabine-based chemotherapy repressed MYC proto-oncogene (MYC) and triggered a senescence-like phenotype, associated with persistence and retention of leukemia repopulating potential, and the transcriptional profile of a P53-independent, NFκB-mediated senescence-associated secretory phenotype [7]. Further, human leukemia propagation after cytarabine and doxorubicin chemotherapy was mediated by a rare quiescent label-retaining cell (LRC) population that was undetectable with standard immunophenotypic markers. AML quiescence was reversible and preserved genetic clonal competition and epigenetic inheritance. LRC quiescence was defined by distinct promoter-centered chromatin and gene expression dynamics controlled by the activator protein-1 (AP-1)/E26 transformation specific (ETS) transcription factor network, where JUN is necessary and sufficient for LRC quiescence and associated with persistence and chemotherapy resistance in diverse patients: single-cell analysis confirmed the hallmark tumor necrosis factor (TNF) signaling via NFκB in LRC and the repression of MYC targets [8].

Evolution of AML cells toward developing drug resistance, and eventually to relapse, involves convergence of their chromatin compaction status toward a pattern that is distinctive of “stem-like” cells, which is conducive to increased expression of NFκB-activated genes [9]. This phenomenon develops irrespective of the genetic mutations carried by AML clones [10].

Multiple lines of evidence, therefore, suggest that relapsed and refractory leukemia develops through alterations in chromatin, which perturb the normal cellular regulation of inflammatory and stress responses. In particular, during the development of acute leukemia phenotypes that resist drug treatment and relapse, malignant clones carrying different genetic alterations often converge into specific phenotypes with the apparent criterion being shared patterns of chromatin exposure [11]. These shared patterns include genes involved in innate immune pathways that provide malignant cells with a selective advantage [12]. Such a selective advantage is provided also by expression of cytosolic retinaldehyde dehydrogenases such as ALDH1A1, which was shown to characterize AML cells driving relapse, and which are resistant to chemotherapy [1, 13]. 

Therefore, it can be expected that leukemia cells, under all conditions, do not rely on the selection of clones with a specific fixed phenotype to survive chemotherapy and ultimately relapse. In the study by Leonetti et al., the apparent criterion for malignant clone survival after anthracycline-based chemotherapy was the capacity of these cells to activate expression of essential drug resistance genes, such as ALDH1A1, in response to treatment (Fig. 1). Anthracyclines induce DNA double-strand breaks, triggering signaling pathways that activate p53 to promote cell death. However, cell stress simultaneously activates NFκB, which drives the expression of a variety of anti-apoptotic proteins and induces chemoresistance. Regulatory gene mutations are not the sole factor deciding the outcome of NFκB-p53 antagonism in cell fate determination. Chromatin modifications substantially contribute to the final outcome with respect to precise cell fate.

**Fig. 1:**
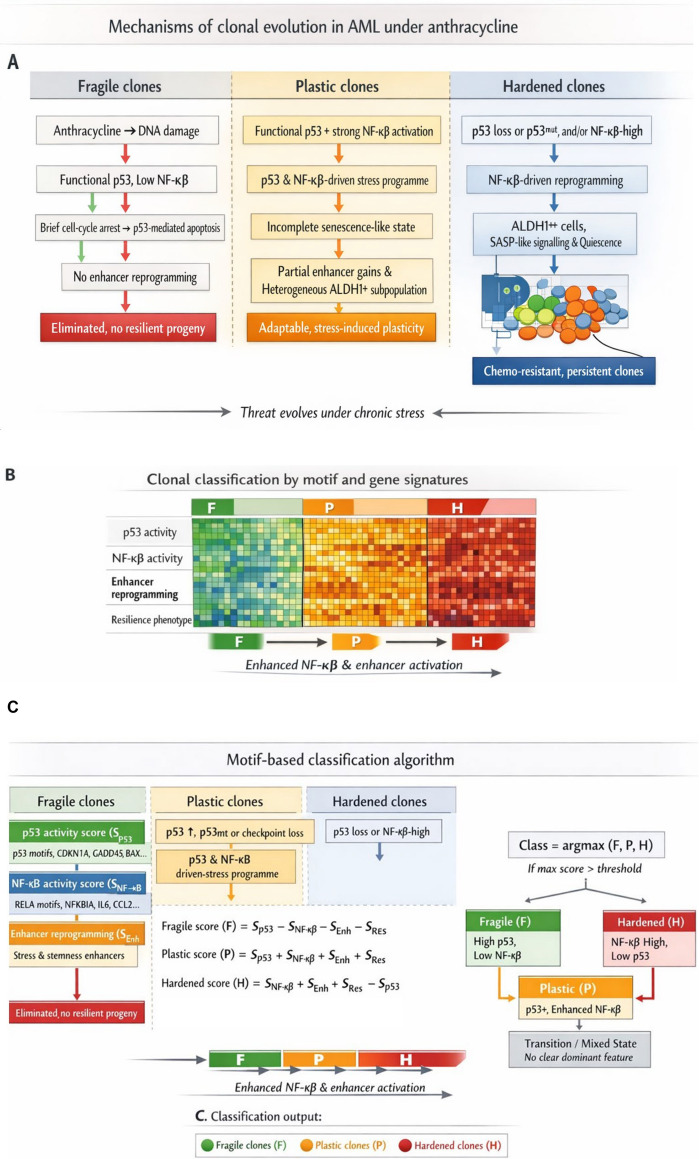
NFκB–driven clonal resilience under anthracycline in AML, from mechanism to classifier. **A** Mechanistic model of clonal evolution under anthracycline-based chemotherapy. Anthracycline-induced DNA damage acts on heterogeneous AML clones that differ in p53 status and NFκB activity (written here as “NF-κβ”). *Fragile clones* (left) are p53-competent with low NFκB; they undergo p53-mediated apoptosis or transient arrest without enhancer reprogramming, leading to elimination and no resilient progeny. *Plastic clones* (middle) retain functional p53 but experience strong NFκB activation (e.g. stress, cytokines, SASP), entering an incomplete senescence-like state with partial gain of stress/stemness enhancers and a mixed ALDH1 + subpopulation, and conferring stress-induced plasticity. *Hardened clones* (right) have p53 loss and/or high NFκB, undergo NFκB–driven enhancer reprogramming, acquire ALDH1 + + cells, SASP and quiescence, and persist as chemo-resistant clones. The overarching concept is that “threat evolves under chronic stress.” **B** Clonal classification by motif and gene signatures. Clones (cells, subclones, or samples) are stratified into *Fragile (F)*, *Plastic (P)*, and *Hardened (H)* states using four feature blocks: p53 activity, NFκB activity, enhancer reprogramming, and resilience phenotype. The heatmap illustrates low NFκB/enhancer/resilience and high p53 in Fragile clones, intermediate activation of all four blocks in Plastic clones, and high NFκB/enhancer/resilience with reduced p53 in Hardened clones. Arrows from F → P → H indicate a potential trajectory under sustained therapy-induced stress, driven by increasing NFκB signaling and enhancer activation. **C** Motif-based classification algorithm. Four input scores are computed per clone: p53 activity ((S_{p53})), NFκB activity ((S_{NF\kappa B})), enhancer reprogramming ((S_{enh})), and resilience phenotype ((S_{res})). These are combined into three composite scores: Fragile (F = S_{p53}—(S_{NF\kappa B} + S_{enh} + S_{res})), Plastic (P = S_{p53} + S_{NF\kappa B} + S_{enh} + S_{res}), and Hardened (H = S_{NF\kappa B} + S_{enh} + S_{res}—S_{p53}). The class is assigned as (\text{argmax}(F, P, H)), with an optional threshold to define confident calls; ambiguous cases are labeled as transition/mixed states. This panel links the conceptual model to a concrete, motif- and signature-based classifier that can be applied to bulk or single-cell datasets. The author used Microsoft Copilot to assist with figure drafting and visualization. The tool supported the organization of conceptual elements and the preparation of schematic artwork. All scientific content, interpretation, and final figure design were developed and verified solely by the author in aggregate rounds of editing

Inhibitors of cytosolic ALDH1 enzymes, such as ALDH1A1, ALDH1A2, and ABD-3001, are currently in clinical trial in a Phase I/II study designed to evaluate their safety, tolerability, pharmacokinetics, and pharmacodynamics, in patients with AML who have relapsed or are refractory to standard treatments (NCT05601726). In mice xenografted with human AML, the ABD-3001 active agent DIMATE specifically eradicated human AML cells (hCD45 +) in blood, spleen, and bone marrow in vivo, while sparing healthy circulating mouse cells (mCD45 +) [14]. This inhibitor may also prove useful in treating glucocorticoid-resistant T-cell acute lymphoblastic leukemia [15].

As research increasingly uncovers the genes regulated by drug-induced and stress-induced chromatin alterations, we expect a better picture to emerge soon, regarding the various types of underlying mechanisms that trigger leukemia progression. The findings discussed here strongly support an integrated approach that in addition to the mutation status of relapse-associated malignant cells, takes into consideration the patterns of exposure and chromatin modifications.

## Data Availability

No datasets were generated or analysed during the current study.
